# Microbial Consortia: An Engineering Tool to Suppress Clubroot of Chinese Cabbage by Changing the Rhizosphere Bacterial Community Composition

**DOI:** 10.3390/biology11060918

**Published:** 2022-06-15

**Authors:** Jinhao Zhang, Waqar Ahmed, Zhenlin Dai, Xinghai Zhou, Zulei He, Lanfang Wei, Guanghai Ji

**Affiliations:** 1 State Key Laboratory for Conservation and Utilization of Bio-resources in Yunnan, Yunnan Agricultural University, Kunming 650201, China; jinhaoynau@163.com (J.Z.); ahmed.waqar1083@yahoo.com (W.A.); zhenlin.live@foxmail.com (Z.D.); xinghaizhou2022@163.com (X.Z.); zuleihe@163.com (Z.H.); 2 Key Laboratory of Agro-Biodiversity and Pest Management of Ministry of Education, Yunnan Agricultural University, Kunming 650201, China; 3 Agricultural Foundation Experiment Teaching Center, Yunnan Agricultural University, Kunming 650201, China

**Keywords:** *Plasmodiophora brassicae*, rhizosphere bacterial community, biological control, microbial consortia, disease incidence, high-throughput sequencing

## Abstract

**Simple Summary:**

Clubroot, caused by *Plasmodiophora brassicae* Woron, seriously affects cruciferous plants. The pathogen survives in the soil in the form of resting spores and infects crops for many generations. Currently, clubroot management relies on pesticides and resistant varieties with several limitations. Therefore, there is a need to devise environmentally friendly control measures to mitigate this devastating pathogen. The occurrence and damage of clubroot can be alleviated by microbial consortia. The biocontrol potential of *Bacillus cereus* BT-23, *Lysobacter antibioticus* 13-6, and *Lysobacter capsici* ZST1-2 was investigated as sole strains, intra-/inter-genus co-cultures, and microbial consortia for clubroot disease, plant growth, and rhizosphere bacterial diversity in a field experiment. The microbial consortia efficiently controlled the disease incidence with a successful biocontrol effect of about 65.78%, reshaped the rhizosphere’s microbial diversity, and reduced the soil acidity. Thus, we conclude that microbial consortia suppress the disease incidence by recovering the imbalance in the indigenous microbial community composition. This study highlights the potential of microbial consortia as an engineering tool to control soilborne diseases by reshaping the rhizosphere microbiome.

**Abstract:**

Clubroot disease, caused by *Plasmodiophora brassicae*, is a serious threat to Chinese cabbage (*Brassica rapa* subsp. *pekinensis*) production, which results in extensive yield losses. At present, clubroot control mainly depends upon pesticides, which provoke food-safety concerns, and the application of sole biocontrol agents cannot successfully control the disease. In this study, we investigated the effect of *Bacillus cereus* BT-23, *Lysobacter antibioticus* 13-6, and *Lysobacter capsici* ZST1-2 as sole strains, intra-/inter-genus co-culture, and microbial consortia on clubroot disease, plant growth, and rhizosphere bacterial diversity in a field experiment. The microbial consortia efficiently controlled the incidence of clubroot disease, with a biocontrol effect of about 65.78%, by decreasing the soil acidity and enhancing the yield (17,662.49 kg/acre). The high-throughput sequencing results demonstrated that the phyla Proteobacteria and Bacteroidetes were present in high relative abundance in the rhizosphere soil of the Chinese cabbage. Furthermore, Firmicutes was found as a unique phylum in the rhizosphere soil of CK-H and T1-T7, except for CK-D. The application of microbial consortia recovers the imbalance in indigenous microbial communities. Therefore, we conclude that microbial consortia can reduce the clubroot incidence in Chinese cabbage by decreasing the soil acidity and altering the diversity and structure of rhizosphere bacterial communities. This study highlights the potential of microbial consortia as an engineering tool to control devastating soilborne diseases in commercial crops.

## 1. Introduction

Clubroot disease is caused by *Plasmodiophora brassicae* Woron, an obligate biotrophic pathogen belonging to the kingdom Protista. It is an emerging threat to the production of the *Brassicaceae* family crop worldwide, including China [1]. *P. brassicae* has a broad host range, infecting more than 300 species of cruciferous plants, and is widely distributed in more than 60 countries, resulting in 10 to 15% yield loss around the globe [2]. The incidence of clubroot disease is reported all over China, with the average yield loss ranging from 20 to 30% [3]; whereas the regions of Chongqing, Hubei, Sichuan, Hunan, Yunnan, and Zhejiang are most affected by this disease and under a severe threat of yield reduction [3].

*P. brassicae* displays a very complex life cycle consisting of different zoosporic stages, the formation of plasmodia inside host cells, and resting spores [4]. Diseased soil acts as a primary source of infection, and the pathogen survives in the soil (up to 20 years) as resting spores [5]. During the growing season of host plants, the zoospores germinate from the resting spores and enter the host plants through wounds and root hairs, which causes cell swelling. The pathogen multiplies in the cortex tissues and results in “gall or club” formation on the roots, which inhibits the plants’ water- and nutrient-uptake ability [6]. Infected plants generally show symptoms of yellowing, stunted growth, and wilting, leading to the death of the whole plant [6]. Once a pathogen completes its life cycle within a host plant, millions of zoospores are released from the galls to the rhizosphere soil and spread from plant to plant, within fields, and from field to field through irrigation water, mechanical operations, or water erosion [3].

The prevention and control of cruciferous clubroot have become foremost concerns due to the broad host range and long-term persistence in the soil. Currently, the main method to control this disease is the application of fungicides, such as benzimidazoles, chlorothalonil, cyclophosphamide, and quintozene [3], resistant cultivars [7], crop rotation [8], and liming [9]. Many studies have reported that the excessive use of agrochemicals develops resistance in the pathogen and causes human-health concerns [10,11]. The resistant varieties may also lose their resistance against the pathogen due to the rapid mutation ability and strong pathogenicity of *P. brassicae* [12]. It is suggested that these methods have limitations and can only reduce the incidence of clubroot disease, not eradicate it. Therefore, there is an urgent need to develop durable, efficient, and environmentally friendly control measures to mitigate this devastating disease.

Currently, biological control through potent endophytes and rhizobacteria provides an effective and environmentally friendly solution to control the incidence of soilborne field-crop diseases [13]. Previous studies demonstrated that biocontrol agents (BCAs) suppress soilborne diseases through the mechanism of antibiosis, niche exclusion, nutrient acquisition, the induction of resistance, plant-growth promotion, and the production of antimicrobial compounds [14,15,16]. Liu et al. reported that *Bacillus subtilis* XF-1 can produce antimicrobial compounds and suppress the incidence of clubroot disease up to 76.92% by improving the soil microbial diversity [17]. The application of *Streptomyces alfalfae* XY25*^T^* improves soil health, regulates rhizospheric bacterial and fungal communities, enhances plant growth, and mitigates clubroot disease by up to 69.4% [11]. *B. subtilis* QST713 can reduce clubroot incidence in canola crops by up to 86% through the induction of host resistance and antibiosis [18]. Similarly, it is reported that *B. cereus* BT-23, *L. antibioticus* 13-6, and *L. capsici* ZST1-2 effectively mitigate the incidence of clubroot and root rot on Chinese cabbage and *Panax notoginseng*, respectively, and also act as plant-growth promoters [1,19].

The rhizosphere is the narrow region of soil or substrate that is directly influenced by root secretions and associated soil microorganisms, known as the root microbiome [20]. The plant rhizosphere acts as a major habitat for the diversity of microorganisms and is considered one of the most complex ecosystems on earth [20]. Many studies reported that an imbalance in rhizospheric microbial diversity results in the development of soilborne diseases [19,21]. The improper use of agrochemicals leads to imbalances in the rhizosphere environment, affects soil health, and influences survival and growth of pathogens [22]. Therefore, soil health and a diverse community of beneficial microbes are considered the key factors for healthy crop production and disease suppression [19,23]. Bacterial consortia have been reported to improve beneficial traits in plants compared to individual strains due to the coverage of a diverse set of plant-growth-promotion and biological control mechanisms [24]. The use of microbial consortia is a practical approach for ameliorating nutrient uptake, salinity, drought, and phytopathogenic infections of crops [25]. Furthermore, some bacterial consortia can fix nitrogen, convert some unavailable nutrients into soluble forms, produce phytohormones, and chelate iron, all of which are important for maintaining soil health and can help to minimize the negative impact of some non-sustainable agricultural practices [24,25].

In our previous study, we found that inter-genus bacterial co-culture produced more secondary metabolites and significantly suppressed clubroot disease incidence compared with intra-genus bacterial co-culture and single strains [1]. However, their effect on rhizospheric bacterial community diversity is still unknown. This study enhances our knowledge to decipher the impact of these biocontrol agents on soil health and bacterial community diversity when applied as single, inter-/intra-genus co-culture, and microbial consortia. The study aimed to provide theoretical and experimental knowledge to explore the relationship between rhizospheric bacterial community diversity and *P. brassicae* to mitigate clubroot by engineering the rhizosphere microbiome.

## 2. Materials and Methods

### 2.1. Biocontrol Bacterial Strains, Growth Medium, and Culture Conditions

The biocontrol strains *Bacillus cereus* BT-23, *Lysobacter antibioticus* 13-6, and *Lysoabcter capsici* ZST1-2 used in this study were preserved in our laboratory. *B. cereus* BT-23 was isolated from the rhizosphere soil of healthy Chinese cabbage plant, and *L. antibioticus* 13-6 and *L. capsica* ZST1-2 were isolated from rhizosphere soil of healthy Konjac plant and purple potato, respectively. Before the start of each experiment, bacterial strains were grown on King’s B (KB) agar medium (Peptone 20 g∙L^−1^, KH_2_PO_4_ 1.5 g∙L^−1^, MgSO_4_·7H_2_O 1.5 g∙L^−1^, Glycerol 10 mL∙L^−1^, Agar 20 g∙L^−1^, and pH 7.0) at 28 ℃ for 48 h [1]. The pure culture of bacterial strains was stored in 50% (*v*/*v*) glycerol solution at −80 ℃ for future use.

### 2.2. Assembly of Microbial Consortia

A single colony was picked from the pure culture, transferred into 500 mL of KB broth, and cultured overnight at 28 °C and 160 rpm to obtain three simplified probiotic complexes, as follows: (i) single strains (*B. cereus* BT-23, *L. antibioticus* 13-6, *L. capsici* ZST1-2), (ii) inter-/intra-genus co-culture (*B. cereus* BT-23 + *L. antibioticus* 13-6, and *L. antibioticus* 13-6 + *L. capsici* ZST1-2), and (iii) microbial consortia (*B. cereus* BT-23 + *L. antibioticus* 13-6 + *L. capsici* ZST1-2). The optical density of culture mediums was adjusted to the same degree OD_600 nm_ = 0.5 using a spectrophotometer [1].

### 2.3. Experimental Site, Experimental Conditions, and Design Descriptions

A field experiment was conducted to investigate the biocontrol effect of potential strains on clubroot using Chinese cabbage variety 83-1 (highly susceptible) during the growing season in April–June 2020 in Dabai County, Panlong District, Kunming City (25°2′47.04″ N and 102°42′33.84″ E), Yunnan Province, China. Chinese cabbage has been continuously growing in this field for the last 10 years and is highly infected with *P. brassicae*. The average annual temperature is about 15.1 °C, and total rainfall is about 1534 mm per year. Seedlings of Chinese cabbage variety 83-1 (highly susceptible) and Kangda No. 3 (highly resistant) were provided by the Qingdao International Seed Co., LTD (Shandong, China) and Yunnan Academy of Agricultural Sciences (Yunnan, China), respectively.

The experiment was performed under 9 conditions as follows: untreated susceptible variety 83-1 as a disease control group (CK-D), untreated resistant variety Kangda No. 3 as a healthy control group (CK-H), single-strain *L. antibioticus* 13-6(T1), single-strain *L. capsici* ZST1-2 (T2), single-strain *B. cereus* BT-23 (T3), intra-genus co-culture of *L. antibioticus* 13-6 + *L. capsici* ZST1-2 (T4), inter-genus co-culture of *L. antibioticus* 13-6 + *B. cereus* BT-23 (T5), microbial consortia of *B. cereus* BT-23 + *L. antibioticus* 13-6 + *L. capsici* ZST1-2 (T6), and commercial fungicide Kejia (Ishihara Sangyo Kaisha, Ltd., Osaka, Japan) (T7). Seedlings of Chinese cabbage were transplanted in a plot (1.8 m × 8 m) on ridges with a plant-row distance of 40 cm × 40 cm in a randomized complete block design. One week after transplantation, treatments T1–T6 and T7 were inoculated thrice with 250 mL/plot of bacterial suspension and 80–100 mL/hm^2^ commercial fungicide Kejia, respectively, with an interval of 7 days, through soil drenching method with irrigation water. The experiment was performed in replicates with 3 plots per treatment and 80 plants per plot.

### 2.4. Assessment of Biocontrol Effect, Plant Growth Promotion, and Soil pH

The biocontrol and plant growth promotion potential of single strains, inter-/intra-genus co-culture, and microbial consortia of bacterial strains were examined after 2 months of transplantation in June 2020. Randomly, 30 plants per plot were uprooted to determine the biocontrol potential of biocontrol strains, whereas plant growth promotion ability was calculated as the fresh weight of plants without roots in terms of yield (kg/acre). Soil pH was measured using a pH meter in a 1:2.5 soil/water (W/V) suspension. The disease index was investigated using a 5-point disease graded scale, as follows; 0 = no gall, 1 = main root swollen and its diameter less than 2 times the base of the stem, 3 = main root swollen and its diameter 2 to 3 times the stem base, 5 = main root swollen and the diameter of the stem base of 3 to 4 times, 7 = main root swollen and its diameter is more than 4 times the base [1]. Disease incidence (Di), disease index (DI), and control effect (C.E) were calculated using the following formulas:

DI = ∑(number of diseased plants × disease grading scale)/(total numbers of investigated plants × highest disease rating scale) × 100

Di (%) = (number of diseased plants/total number of investigated plants) × 100

C.E (%) = (disease index of control − disease index of treatment/disease index of control) × 100

### 2.5. Soil-Sample Collection and Extraction of DNA

Bulk soil was removed from the roots by gently shaking the plants (10 plants per replicate), and soil attached to the root surface was taken as rhizosphere soil samples (3 replicates/treatment). Total soil DNA was extracted from 0.5 g of soil/sample using a PowerSoil^®^ DNA extraction kit (MO BIO Laboratories, Carlsbad, CA, USA). The DNA quality was quantified at OD_260/280 nm_ 1.7–1.9 using a NanoDrop spectrophotometer (ND2000, Thermo Scientific, Waltham, MA, USA) [19], and extracted DNA was kept at −20 °C for further studies.

### 2.6. PCR Amplification and Analysis of Rhizospheric Bacterial Diversity

The V3–V4 regions of the 16S rRNA gene were amplified using primer pair 343F (5′-TACGGRAGGCAGCAG-3′) and 798R (5′- AGGGTATCTATCCT-3′) to explore the rhizosphere bacterial diversity [26]. The PCR products were purified through Nextera XT Index PCR clean-up kit (Illumina Inc., San Diego, CA, USA) and sequenced on an Illumina MiSeq platform at OE Biotech Co., Ltd. (Shanghai, China). Raw data collected from the Illumina sequence were processed through UCHIME (Version 8.1) to remove Chimeras [27] and quality-controlled at a 20% cutoff level using Trimmomatic software (Version 0.33) to generate clean reads [28]. The clean reads were then clustered into operational taxonomic units (OTUs) at a 3% dissimilarity level through UPARSE [29] and blasted against Ribosomal Database Project (RDP) classifier in the SLIVA database (http://www.arb-silva.de) of bacteria for taxonomic annotation [30].

### 2.7. Bioinformatics Analysis

Alpha diversity indices (OTUs, Chao 1, Shannon, and Simpson) and beta diversity indices based on Bray–Curtis dissimilarity matrix under different treatments were calculated using QIIME 2. Principal coordinate analysis (PCoA) based on Bray–Curtis dissimilarity matrix was used to visualize the changes in bacterial community structure. The relative-abundance bar plots at the phyla and genus levels were generated using R scripts in R v3.5.3. Correlation analyses were performed according to Pearson correlation coefficient (PCC, *p* < 0.05) at genera level and alpha diversity indices between disease index, yield, and soil pH using the HMISC package in R and visualized through a heatmap. Data were statistically analyzed using analysis of variance (ANOVA) at *p <* 0.05, and means were compared using a *t*-test and Duncan’s multiple range test at *p <* 0.05.

## 3. Results

### 3.1. Assessment of Disease Incidence and Soil pH

**Figure 1:**
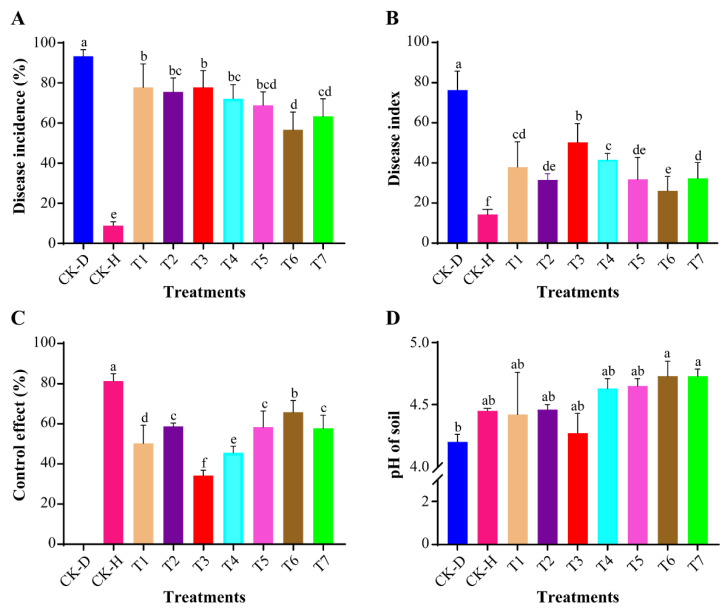
Biocontrol potential of candidate biocontrol strains as single strains, intra/inter-genus co-culture, and microbial consortia against clubroot disease of Chinese cabbage. Disease incidence (**A**), disease index (**B**), control effect (**C**), and pH (**D**). Different small letters on the error bars are shown the significant difference among treatments according to Duncan’s multiple range test at *p* < 0.05. Disease control (CK-D), healthy control (CK-H), single-strain *Lysobacter antibioticus* 13-6(T1), single-strain *L. capsici* ZST1-2 (T2), single-strain *Bacillus cereus* BT-23 (T3), intra-genus co-culture of *L. antibioticus* 13-6 + *L. capsici* ZST1-2 (T4), inter-genus co-culture of *L. antibioticus* 13-6 + *B. cereus* BT-23 (T5), microbial consortia *B. cereus* BT-23 + *L. antibioticus* 13-6 + *L. capsici* ZST1-2 (T6), and commercial fungicide Kejia (T7).

At the end of the experiment, we assessed the effect of bacterial biocontrol strains as single strains, inter-/intra-genus, and microbial consortia on clubroot disease and soil pH (Figure 1 and Table S1). Results demonstrated that the Chinese cabbage variety Kangda No. 3 (CK-H) showed resistance against *P. brassicae* with minimum disease incidence (8.89%) and maximum control effect (81.27%). The application of microbial consortia (T6) suppressed the incidence of clubroot disease, displaying a control effect of about 65.78% compared with commercial fungicide Kejia (T7: 57.66%), single strains (T1: 50.23%, T2: 58.69%, and T3: 34.19%), and inter-/intra-genus co-cultures (T4:45.54% and T5: 58.32%) of bacterial biocontrol strains (Figure 1A–C). The pH of Chinese cabbage rhizosphere soil significantly increased under treatments T1–T7 and healthy control (CK-H) compared to disease control (CK-D). Soil pH increased up to 12.62% under T6 and T7 compared with disease control (CK-D) (Figure 1D and Table S1).

### 3.2. Evaluation of Plant Growth Promotion Potential of Biocontrol Strains

**Figure 2:**
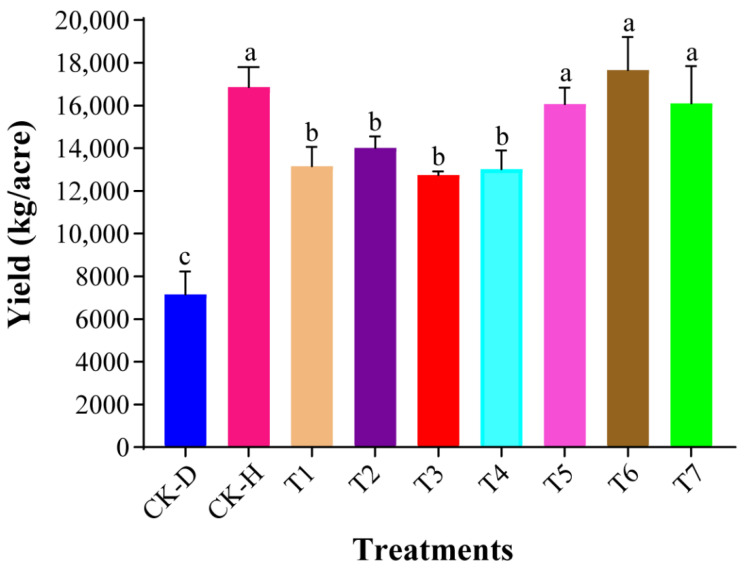
Plant-growth promotion potential of candidate biocontrol strains as single strains, intra/inter-genus co-culture, and microbial consortia on Chinese cabbage. Significant differences between treatments are shown by different small letters on the error bars according to Duncan’s multiple-range test at *p* < 0.05. Disease control (CK-D), healthy control (CK-H), single-strain *Lysobacter antibioticus* 13-6 (T1), single-strain *L. capsici* ZST1-2 (T2), single-strain *Bacillus cereus* BT-23 (T3), intra-genus co-culture of *L. antibioticus* 13-6 + *L. capsici* ZST1-2 (T4), inter-genus co-culture of *L. antibioticus* 13-6 + *B. cereus* BT-23 (T5), microbial consortia *B. cereus* BT-23 + *L. antibioticus* 13-6 + *L. capsici* ZST1-2 (T6), and commercial fungicide Kejia (T7).

We investigated the plant-growth promotion potential of bacterial biocontrol strains in terms of yield (kg/acre) as single strains, inter-/intra-genus co-culture, and microbial consortia and compared them with the disease control (CK-D), healthy control (CK-H), and commercial fungicide (T7)**.** The results revealed that the yield (kg/acre) of the Chinese cabbage significantly increased under the healthy control (CK-H), the inter-genus co-culture of *L. antibioticus* 13-6 + *B. cereus* BT-23 (T5), the microbial consortia (T6), and the commercial fungicide (T7) compared with the disease control (CK-D), single strains (T1–T3), and intra-genus co-culture (T4) (Figure 2 and Table S2). The highest yield (17,662.49 kg/acre) was recorded when treated with microbial consortia (T6), compared with the disease control (CK-D), healthy control (CK-H), and other treatments (T1–T5 and T7). The yield (kg/acre) of Chinese cabbage treated with microbial consortia (T6), commercial fungicide (T7), and healthy control (CK-H) increased up to 146.50%, 124.66%, and 135.51%, respectively, compared with the disease control (CK-D). When the biocontrol agents were applied as single strains and inter-/intra-genus co-culture, the yield (kg/acre) of the Chinese cabbage treated with single-strain *L. capsici* ZST1-2 (T2) and inter-genus co-culture *B. cereus* BT-23 + *L. antibioticus* 13-6(T5) significantly increased compared to the other single strains (T1 and T3) and intra-genus co-culture (T4). The yield (kg/acre) of the Chinese cabbage treated with T2 and T5 increased up to 95.66% and 124.28% compared with the disease control (CK-D).

### 3.3. Assessment of Alpha Diversity of Bacterial Community Associated with Rhizosphere Soil of Chinese Cabbage

The alpha diversity indices (OTUs at >97%, Chao 1, Shannon, and Simpson indexes) of the bacterial community in the rhizosphere soil of the Chinese cabbage are summarized in Table 1 and Table S3. The diversity indices Chao 1, Shannon, Observed_species, and PD_whole_tree significantly changed under different treatments but had no impact on the Goods_coverage and Simpson indexes. The alpha diversity indices of the treatments (T1–T7) and the healthy control (CK-H) were found to be significantly higher than the disease control (CK-D), whereas no significant difference was observed between the treatments (T1-T7) and the healthy control (CK-H). By comparing the alpha diversity indices of the bacterial community under different treatments of bacterial biocontrol strains, we found that the Chao 1, Shannon, Observed_species, and PD_whole_tree indices of the microbial consortia (T6) and intra-genus co-culture (T4) were higher than those of single strains (T1-T3), inter-genus co-culture (T5), and commercial fungicide (T7).

### 3.4. Investigation of Bacterial Community Structure in the Rhizosphere Soil of Chinese Cabbage

**Figure 3:**
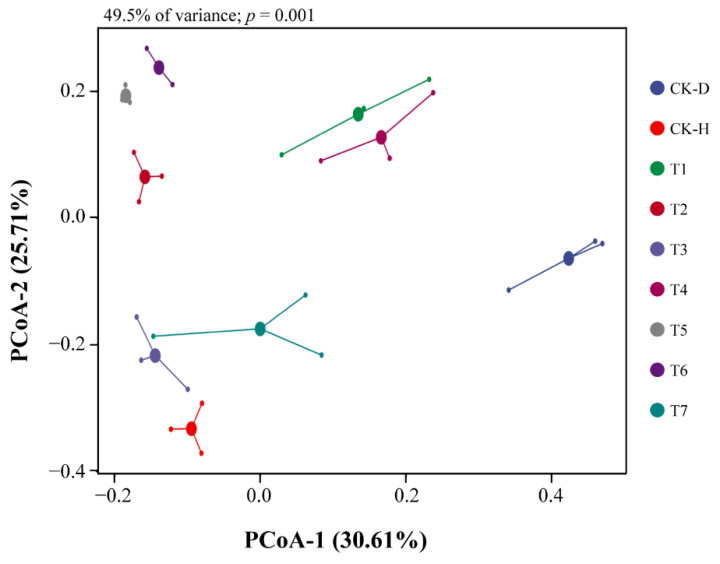
Principal coordinate analysis (PCoA) based on the Bray–Curtis dissimilarity metric showing the beta diversity of bacterial community structure under different treatments (n = 3/treatment). Disease control (CK-D), healthy control (CK-H), single-strain *Lysobacter antibioticus* 13-6 (T1), single-strain *L. capsici* ZST1-2 (T2), single-strain *Bacillus cereus* BT-23 (T3), intra-genus co-culture of *L. antibioticus* 13-6 + *L. capsici* ZST1-2 (T4), inter-genus co-culture of *L. antibioticus* 13-6 + *B. cereus* BT-23 (T5), microbial consortia *B. cereus* BT-23 + *L. antibioticus* 13-6 + *L. capsici* ZST1-2 (T6), and commercial fungicide Kejia (T7).

The principal coordinate analysis (PCoA) based on the Bray–Curtis dissimilarity matrix showed that the structure of the bacterial communities in the rhizosphere of Chinese cabbage significantly changed under different treatments (Figure 3). By comparing the structure of the rhizosphere bacterial communities under different treatments, we found that the treatment groups (T1–T7) and the healthy control group (CK-H) were significantly separated from the disease control (CK-D). According to PCoA results, the first two axes explain 30.61 and 25.71% of the total variation in the bacterial community structure under different treatments (analysis of variance 49.5% and *p* < 0.001) (Figure 3).

### 3.5. Analysis of Bacterial Community Composition at the Phylum Level

**Figure 4:**
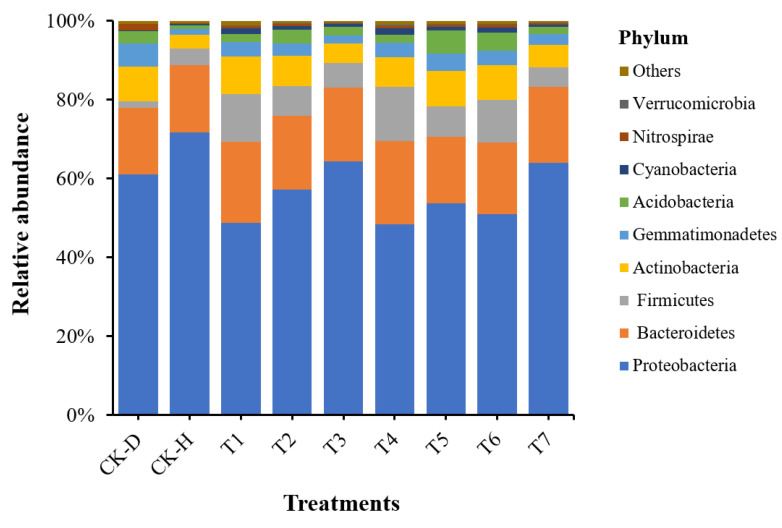
Relative abundance bar plots for 10 most dominant bacterial phyla under different treatments (n = 3/treatment). Disease control (CK-D), healthy control (CK-H), single-strain *Lysobacter antibioticus* 13-6 (T1), single-strain *L. capsici* ZST1-2 (T2), single-strain *Bacillus cereus* BT-23 (T3), intra-genus co-culture of *L. antibioticus* 13-6 + L. capsici ZST1-2 (T4), inter-genus co-culture of *L. antibioticus* 13-6 + *B. cereus* BT-23 (T5), microbial consortia *B. cereus* BT-23 + *L. antibioticus* 13-6 + *L. capsici* ZST1-2 (T6), and commercial fungicide Kejia (T7).

The relative abundance analysis at the phylum level showed that the bacterial community composition significantly changed under the different treatments. The relative abundance bar plots for the 10 most dominant bacterial phyla are shown in Figure 4. In all the rhizosphere soil samples, the dominant bacterial phyla were Proteobacteria, Bacteroidetes, and Firmicutes (average abundance: 57.80%, 18.59%, and 7.68%, respectively), followed by Actinobacteria (7.29%), Gemmatimonades (3.44%), Acidobacteria (2.86%), Cyanobacteria (0.90%), Nitrospirae (0.57%), Verrucomicrobia (0.18%), and others (0.69%). Bacterial phyla, such as Bacteroidetes, Firmicutes, and Cyanobacteria, with CK-H and T1–T7 were present in a high proportion compared to with CK-D. By contrast, the phyla Actinobacteria, Gemmatimonades, Acidobacteria, and Nitrospirae displayed a higher proportion of CK-D than CK-H and T1–T4. However, Proteobacteria was present in a higher relative abundance with CK-H, T3, and T4 than with CK-D (Table S4). *L. antibioticus* 13-6 and *L. capsici* ZST1-2 belong to Proteobacteria, while *B. cereus* 1-BT-23 belongs to Firmicutes. The application of *L. antibioticus* 13-6, *L. capsici* ZST1-2, and *B. cereus* 1-BT-23 as single strains, co-cultures, and microbial consortia enhanced the abundance of Firmicutes in the rhizosphere of the Chinese cabbage compared with CK-D. The abundance of the Proteobacteria decreased in the rhizosphere of the Chinese cabbage treated with *L. antibioticus* 13-6 and *L. capsici* ZST1-2 compared to CK-D and increased when treated with *B. cereus* 1-BT-23. The data related to the 15 highest bacterial taxa in terms of order and family level are shown in Figure S2.

### 3.6. Changes in the Bacterial Community Composition at the Genus Level

**Figure 5:**
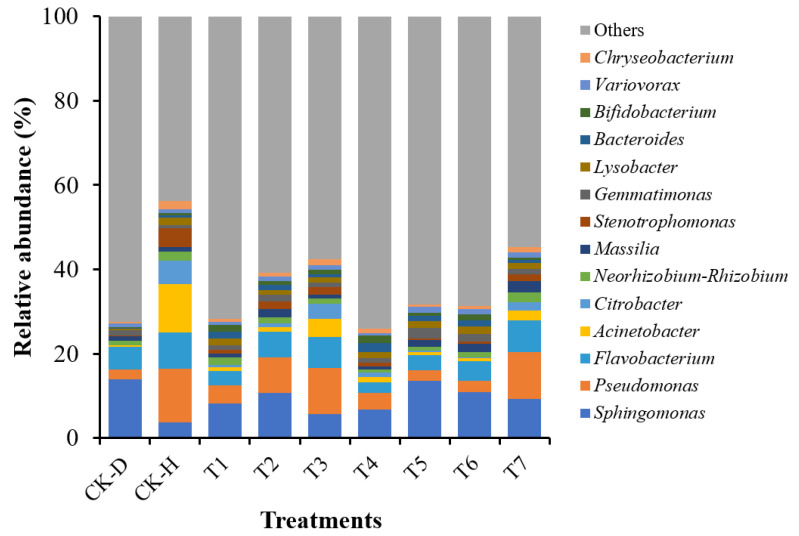
Relative abundance bar plots for 15 most dominant bacterial genera under different treatments (n = 3/treatment). Disease control (CK-D), healthy control (CK-H), single-strain *Lysobacter antibioticus* 13-6 (T1), single-strain *L. capsici* ZST1-2 (T2), single-strain *Bacillus cereus* BT-23 (T3), intra-genus co-culture of *L. antibioticus* 13-6 + *L. capsici* ZST1-2 (T4), inter-genus co-culture of *L. antibioticus* 13-6 + *B. cereus* BT-23 (T5), microbial consortia *B. cereus* BT-23 + *L. antibioticus* 13-6 + ZST1-2 (T6), and commercial fungicide Kejia (T7).

The bacterial community composition significantly changed at the genus level under different treatments. The relative abundance bar plots for the 15 most dominant bacterial genera are shown in Figure 5. The bacterial genera, such as *Sphingomonas*, *Pseudomonas*, *Flavobacterium*, *Acinetobacter*, and *Citrobacter*, were present in high relative abundance (average abundance: 9.21%, 6.52%, 5.46%, 2.57%, and 1.58%, respectively) in all the rhizosphere soil samples. However, the genus *Sphingomonas* was present in a higher relative abundance (14.02%) in the rhizosphere soil of the disease control group (CK-D) compared to the healthy control group (CK-H) and the other treatments (T1–T7). The genus *Pseudomonas* was present in a higher relative abundance in the rhizosphere soil of the healthy control group (CK-H) and other treatments (T1–T7) than the disease control group (CK-D) (Table S5). These results suggested that the high relative abundance of the *Pseudomonas* and *Sphingomonas* genera in the rhizosphere soil of the healthy (CK-H) and diseased (CK-D) control groups played an important role in disease suppression and progression, respectively. The abundance of the genus *Lysobacter* was also increased in the rhizosphere soil of all the treatment groups, except for disease control (CK-D). Whereas the genus *Bacillus* was present in the same abundance in the rhizosphere soil of all the treatment groups, and no difference was observed among the treatments (Table S5). 

### 3.7. Correlation Analysis of Disease Index, Yield, and pH between Bacterial Genera and Diversity Indices

**Figure 6:**
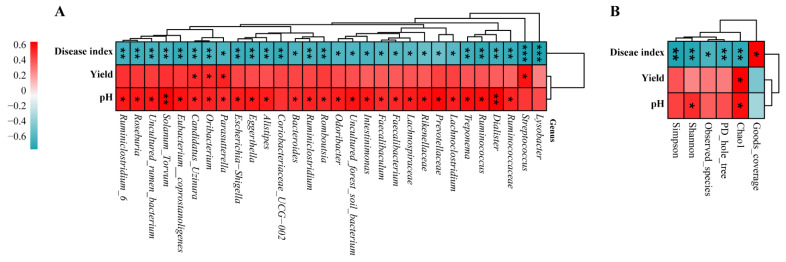
Correlation analysis of disease index, yield, and pH between bacterial genera and diversity indices. Correlation analysis of disease index, yield, and pH between bacterial genera (**A**) and correlation analysis of disease index, yield, and pH between alpha diversity indices (**B**). Asterisks represents the significant differences among treatments according to *t*-test at * *p* < 0.05, ** *p* < 0.01, and *** *p* < 0.001.

The heatmaps generated for the correlation analyses of the disease index, yield, and pH between the bacterial genera and the diversity indices are shown in Figure 6. The bacterial genera, such as *Lysobacter*, *Streptococcus*, *Ruminococcaceae*, *Dialister*, *Ruminococcus*, *Treponema*, *Lachnoclostridium*, *Prevotellaceae*, *Rikenellaceae*, *Lachnospiraceae*, *Faecalibacterium*, *Faecalibaculum*, *Intestinimonas*, *Odoribacter*, *Romboutsia*, *Ruminiclostridium*, *Bacteroides*, *Coriobacteriaceae*, *Alistipes*, *Eggerthella*, *Escherichia−Shigella*, *Parasutterella*, *Oribacterium*, *Candidatus_Uzinura*, *Eubacterium_Coprostanoligenes*, *Solanum_Torvum*, and *Roseburia*, were significantly negatively correlated with the occurrence and severity of clubroot disease. However, they were significantly positively correlated with the pH. By contrast, the bacterial genera, including *Streptococcus*, *Parasutterella*, *Oribacterium*, and *Candidatus_Uzinura*, were significantly positively correlated with the yield of the Chinese cabbage (Figure 6A). The diversity indices, including Chao 1, PD_whole, observed_species, Shannon, and Simpson, were significantly negatively correlated with disease occurrence and severity, whereas they were positively correlated with the yield and pH. However, goods_coverage was significantly positively correlated with disease occurrence and severity and negatively correlated with the yield and pH (Figure 6B). 

## 4. Discussion

Clubroot caused by *P. brassicae* is one of the most destructive soilborne diseases affecting the production of Chinese cabbage all over China. Generally, the disease occurs after 30 days of transplantation, and the yield losses it causes can reach up to 80% in severe outbreaks [31]. Over the past few decades, important disease-management methods in the form of resistant cultivars and chemical control have been adopted, but the results were not fruitful [3,11]. Therefore, biological control through potent endophytes and rhizobacteria is considered a practical approach for treating and preventing clubroot disease [11,32]. The application of a single biocontrol agent (BCAs) cannot efficiently control clubroot disease due to the highly virulent nature of *P. brassicae* [1]. Thus, there is an urgent need to develop a long-lasting and environmentally friendly control measure in the form of microbial consortia to mitigate this devastating disease.

Wei et al., reported that the combined (inter-/intra-genus) application of BCAs suppressed the incidence of clubroot disease significantly better than the application of a single strains [1]. In our study, we found that the inter-genus bacterial co-culture showed a maximum control effect of about 58.32% compared with the intra-genus bacterial co-culture and the single strains. However, the control efficacy was increased up to 65.78% when BCAs were applied as microbial consortia. Srivastava et al. [33] reported that the use of BCAs as microbial consortia significantly enhanced tomato yield and suppressed the incidence of *Fusarium* wilt disease by 20 and 67%, respectively. Our results are similar to previous findings [1,34,35] in that the application of BCAs as microbial consortia limits the competition for resources because different microbes occupy diverse niches in the rhizosphere. However, how the microbial consortia affect the bacterial communities in rhizosphere and clubroot development is still unknown and needs further in-depth studies.

The plant rhizosphere microbiome acts as the first line of defense against soilborne pathogens and plays an indispensable role in maintaining plant health and disease control [20,36,37]. Currently, high-throughput sequencing is employed to decipher the microbial community diversity and composition associated with the plant rhizosphere and phyllosphere [17,21]. In this study, the high-throughput sequencing results proved that the application of microbial consortia reduced the incidence of clubroot in Chinese cabbage by reshaping the diversity and structure of the rhizosphere bacterial communities. Zhang et al. [19] and Hu et al. [38], proved that probiotic consortia significantly reduced the incidence of root rot disease in *Panax notoginseng* and the bacterial wilt of tomato by reshaping the rhizosphere microbiome and colonization in the rhizosphere soil, respectively. Our results are similar to those of previous studies [18,19] in that the bacterial community structure and diversity significantly changed after the application of microbial consortia compared with disease control (CK-D) (Figure 3 and Table 1).

This study demonstrates that Proteobacteria was the most dominant phylum in all the rhizosphere soil samples, with an average abundance of about 57.80%, followed by Bacteroidetes and Firmicutes (average abundance: 18.59% and 7.68%, respectively). Our results are in accordance with the findings of Liu et al. [17], who stated that the rhizosphere soil of Chinese cabbage is enriched in Proteobacteria and Bacteroidetes, as well as the finding, in of previous reports, that Proteobacteria is the most dominant bacterial phylum in the rhizosphere soils of plants [39,40]. In our study, the BCAs *L. antibioticus* 13-6 and *L. capsici* ZST1-2 belonged to Proteobacteria, whereas *B. cereus* 1-BT-23 belonged to Firmicutes. The results of the 16S amplicon sequencing showed that the abundance of Firmicutes increased in the rhizosphere soil of the Chinese cabbage under different treatments. However, the abundance of Proteobacteria was reduced in the rhizosphere soil of the Chinese cabbage in all the treatments (T1, T2, T4, T5, and T6) compared to the disease control (CK-D), except for healthy the control (CK-H) and treatments T3 and T7. This may have been because a facilitative interaction took place between *B. cereus* 1-BT-23 and *Lysobacter* species. By contrast, antagonistic interactions occurred between the *Lysobacter* species (*L. antibioticus* 13-6 and *L. capsici* ZST1-2). These findings (facilitative and antagonistic interactions) are similar to those in the study by Wei et al. [1], who reported that in an inter-genus co-culture assay, *B. cereus* 1-BT-23 enhanced the growth of *L. antibioticus* 13-6, whereas in inter-genus (*B. cereus* 1-BT-23 + *L. capsici* ZST1-2) and intra-genus (*L. antibioticus* 13-6 and *L. capsici* ZST1-2) assays, the strains were antagonistic to each other. This may have been because facilitative interactions take place between compatible microbes, which tend to enhance each other’s growth when grown co-cultured in microbial consortia [34].

It is reported that soil health, fertility, acidity, and enzymatic activity are directly proportional to the rhizosphere microbiome composition [11], which correlates with disease development and unhealthy crop production [41]. The occurrence of soilborne diseases is correlated with soil pH. An increase in soil pH is known to enhance the activity of beneficial microorganisms in the rhizosphere [42,43] and to suppress the incidence of soilborne diseases [44,45]. This study showed that microbial consortia (T6) significantly reduce soil acidity (increasing the soil pH value), resulting in maximum yield and reduced disease development. Liming is considered one of the most important disease management strategies to reduce clubroot disease by increasing the soil pH value [9], while the application of consortia delivers a similar effect. Similarly, Hu et al. [11] reported that the application of *S. alfalfae* XY25^*T*^ suppressed the incidence of clubroot disease in Chinese cabbage by altering the soil fertility and acidity. Previous studies demonstrated that a decrease in soil acidity influences soil enzymatic activities, the diversity of rhizosphere microorganisms, and organic matter formation, resulting in reduced disease development [46]. Thus, we suggest that the application of BCAs as microbial consortia enhances the yield and suppresses the incidence of soilborne diseases by improving the diversity of the rhizosphere microbiome and reducing the soil acidity. However, the impact of microbial consortia on soil organic matter and soil enzymatic activities is unclear and needs further studies.

## 5. Conclusions

In summary, we conclude that microbial consortia can be used as an emerging tool to reduce the clubroot incidence in Chinese cabbage by reducing the soil acidity and to improve the rhizosphere bacterial communities in continuous cropping soil. Altogether, our results revealed that the application of bacterial biocontrol strains as microbial consortia has significant potential to mitigate clubroot disease, compared with using single strains and commercial fungicide. This study provides experimental knowledge on the importance of applying microbial consortia to manage clubroot disease on a large scale. Therefore, the construction of microbial consortia is of great importance in crop management due to its plant-growth promotion and disease-suppression properties.

## Figures and Tables

**Table 1:** Effect of single strains, intra/inter-genus co-culture, and microbial consortia on alpha diversity of bacterial communities associated with the rhizosphere of Chinese cabbage (±SEM, n = 3/treatment).

Treatment	Chao1	Shannon	Observed_Species	PD_Whole_Tree
CK-D	2694.36 ± 159.32 c	6.32 ± 0.24 d	1862.87 ± 108.13 e	72.13 ± 3.26 e
CK-H	3995.2 ± 169.91 a	9.53 ± 0.27 a	3235.27 ± 211.87 a	108.94 ± 7.04 a
T1	3433.88 ± 189.53 ab	8.70 ± 1.08 a	2632.2 ± 201.51 c	92.95 ± 5 a
T2	3453.39 ± 143.99 ab	8.84 ± 0.33 ab	2602.9 ± 126.72 c	91.84 ± 3.61 bc
T3	3089.13 ± 255.56 b	8.06 ± 0.57 c	2310.7 ± 233.38 d	83.7 ± 5.85 d
T4	3619.18 ± 207.86 a	9.46 ± 0.18 a	2858.7 ± 139.39 b	100.42 ± 3.76 a
T5	3411.68 ± 112.85 ab	9.13 ± 0.27 ab	2684.27 ± 117.31 c	94.43 ± 2.49 b
T6	3487.64 ± 127.3 ab	9.23 ± 0.06 a	2655.1 ± 87.67 c	94.33 ± 2.58 b
T7	3467.79 ± 257.75 ab	8.5 ± 0.46 b	2580.17 ± 214.62 cd	90.37 ± 5.3 c

Disease control (CK-D), healthy control (CK-H), single-strain *Lysobacter antibioticus* 13-6 (T1), single-strain *L. capsici* ZST1-2 (T2), single-strain *Bacillus cereus* BT-23 (T3), intra-genus co-culture of *L. antibioticus* 13-6 + *L. capsici* ZST1-2 (T4), inter-genus co-culture of *L. antibioticus* 13-6 + *B. cereus* BT-23 (T5), microbial consortia *B. cereus* BT-23 + *L. antibioticus* 13-6 + *L. capsici* ZST1-2 (T6), and commercial fungicide Kejia (T7). Significant differences among treatments are shown by different small letters within the columns, according to the *t*-test at *p* < 0.05.

## Data Availability

All supporting data related to this manuscript are available in the Supplementary Materials.

## Supplementary Materials

The following supporting information can be downloaded at: https://www.mdpi.com/article/10.3390/biology11060918/s1. Figure S1: Effect of bacterial biocontrol strains as single strains, inter-/intra-genus, and microbial consortia on clubroot disease. Figure S2: Relative abundance bar plots for top 15 most abundant bacterial communities at order and family level. Table S1: Effect of bacterial biocontrol strains on the disease incidence, disease index, control effect, and soil pH of cabbage clubroot. Table S2: Effect of bacterial biocontrol strains on yield of Chinese cabbage. Table S3: Effect of single strains, intra/inter-genus co-culture, and microbial consortia on alpha diversity of bacterial community associated with the rhizosphere of Chinese cabbage (±SEM, n = 3/treatment). Table S4: Relative abundance of 10 most dominant phyla under different experimental conditions (±SEM, n = 3/treatment). Table S5: Relative abundance of 20 most dominant genera in rhizosphere soil under different experimental conditions (±SEM, n = 3/treatment).

## References

[1] Wei L., Yang J., Ahmed W., Xiong X., Liu Q., Huang Q., Ji G. (2021). Unraveling the Association between Metabolic Changes in Inter-Genus and Intra-Genus Bacteria to Mitigate Clubroot Disease of Chinese Cabbage. Agronomy.

[2] Ren L., Xu L., Liu F., Chen K., Sun C., Li J., Fang X. (2016). Host range of *Plasmodiophora brassicae* on cruciferous crops and weeds in China. Plant Dis..

[3] Chai A., Xie X., Shi Y., Li B. (2014). Research status of clubroot (*Plasmodiophora brassicae*) on cruciferous crops in China. Can. J. Plant Pathol..

[4] Kageyama K., Asano T. (2009). Life cycle of *Plasmodiophora brassicae*. J. Plant Growth Regul..

[5] Hwang S.F., Strelkov S.E., Feng J., Gossen B.D., Howard R.J. (2012). *Plasmodiophora brassicae: A* review of an emerging pathogen of the Canadian canola (Brassica napus) crop. Mol. Plant Pathol..

[6] Peng G., Pageau D., Strelkov S.E., Gossen B.D., Hwang S.-F., Lahlali R. (2015). A> 2-year crop rotation reduces resting spores of *Plasmodiophora brassicae* in soil and the impact of clubroot on canola. Eur. J. Agron..

[7] Diederichsen E., Frauen M., Linders E.G., Hatakeyama K., Hirai M. (2009). Status and perspectives of clubroot resistance breeding in crucifer crops. J. Plant Growth Regul..

[8] Yang X.X., Huang X.Q., Wu W.X., Xiang Y.J., Lei D.U., Zhang L., Yong L.I.U. (2020). Effects of different rotation patterns on the occurrence of clubroot disease and diversity of rhizosphere microbes. J. Integr. Agric..

[9] Murakami H., Tsushima S., Kuroyanagi Y., Shishido Y. (2002). Reduction of resting spore density of *Plasmodiophora brassicae* and clubroot disease severity by liming. Soil Sci. Plant Nutr..

[10] Botero A., García C., Gossen B.D., Strelkov S.E., Todd C.D., Bonham-Smith P.C., Pérez-López E. (2019). Clubroot disease in Latin America: Distribution and management strategies. Plant Pathol..

[11] Hu Y., Qiu L., Zhang Z., Liu K., Xia X., Xiong S., Zhao S., Zhao Z., Hu Y., Liang Y. (2021). Control of *Streptomyces alfalfae* XY25^*T*^ Over Clubroot Disease and Its Effect on Rhizosphere Microbial Community in Chinese Cabbage Field Trials. Front. Microbiol..

[12] Strelkov S.E., Hwang S.-F., Manolii V.P., Cao T., Fredua-Agyeman R., Harding M.W., Peng G., Gossen B.D., Mcdonald M.R., Feindel D. (2018). Virulence and pathotype classification of *Plasmodiophora brassicae* populations collected from clubroot resistant canola (*Brassica napus*) in Canada. Can. J. Plant Pathol..

[13] Ahmed W., Yang J., Tan Y., Munir S., Liu Q., Zhang J., Ji G., Zhao Z. (2022). *Ralstonia solanacearum*, a deadly pathogen: Revisiting the bacterial wilt biocontrol practices in tobacco and other Solanaceae. Rhizosphere.

[14] Xu X.-M., Jeffries P., Pautasso M., Jeger M.J. (2011). A numerical study of combined use of two biocontrol agents with different biocontrol mechanisms in controlling foliar pathogens. Phytopathology.

[15] Mendes R., Garbeva P., Raaijmakers J.M. (2013). The rhizosphere microbiome: Significance of plant beneficial, plant pathogenic, and human pathogenic microorganisms. FEMS Microbiol. Rev..

[16] Nguvo K.J., Gao X. (2019). Weapons hidden underneath: Bio-control agents and their potentials to activate plant induced systemic resistance in controlling crop Fusarium diseases. J. Plant Dis. Prot..

[17] Liu C., Yang Z., He P., Munir S., Wu Y., Ho H., He Y. (2018). Deciphering the bacterial and fungal communities in clubroot-affected cabbage rhizosphere treated with *Bacillus subtilis* XF-1. Agric. Ecosyst. Environ..

[18] Lahlali R., Peng G., Gossen B., McGregor L., Yu F., Hynes R., Hwang S., McDonald M., Boyetchko S. (2013). Evidence that the biofungicide Serenade (*Bacillus subtilis*) suppresses clubroot on canola via antibiosis and induced host resistance. Phytopathology.

[19] Zhang J., Wei L., Yang J., Ahmed W., Wang Y., Fu L., Ji G. (2020). Probiotic consortia: Reshaping the rhizospheric microbiome and its role in suppressing root-rot disease of *Panax notoginseng*. Front. Microbiol..

[20] Raaijmakers J.M., Paulitz T.C., Steinberg C., Alabouvette C., Moënne-Loccoz Y. (2009). The rhizosphere: A playground and battlefield for soilborne pathogens and beneficial microorganisms. Plant Soil.

[21] Wei Z., Gu Y., Friman V.-P., Kowalchuk G.A., Xu Y., Shen Q., Jousset A. (2019). Initial soil microbiome composition and functioning predetermine future plant health. Sci. Adv..

[22] Xue C., Ryan Penton C., Zhu C., Chen H., Duan Y., Peng C., Guo S., Ling N., Shen Q. (2018). Alterations in soil fungal community composition and network assemblage structure by different long-term fertilization regimes are correlated to the soil ionome. Biol. Fertil. Soils.

[23] Cai Q., Zhou G., Ahmed W., Cao Y., Zhao M., Li Z., Zhao Z. (2021). Study on the relationship between bacterial wilt and rhizospheric microbial diversity of flue-cured tobacco cultivars. Eur. J. Plant Pathol..

[24] Ju W., Liu L., Fang L., Cui Y., Duan C., Wu H. (2019). Impact of co-inoculation with plant-growth-promoting rhizobacteria and rhizobium on the biochemical responses of alfalfa-soil system in copper contaminated soil. Ecotoxicol. Environ. Saf..

[25] Santoyo G., Guzmán-Guzmán P., Parra-Cota F.I., Santos-Villalobos S.D.L., Orozco-Mosqueda M., Glick B.R. (2021). Plant growth stimulation by microbial consortia. Agronomy.

[26] Ahmed W., Dai Z., Liu Q., Munir S., Yang J., Karunarathna S.C., Li S., Zhang J., Ji G., Zhao Z. (2022). Microbial Cross-Talk: Dissecting the Core Microbiota Associated With Flue-Cured Tobacco (*Nicotiana tabacum*) Plants Under Healthy and Diseased State. Front. Microbiol..

[27] Edgar R.C., Haas B.J., Clemente J.C., Quince C., Knight R. (2011). UCHIME improves sensitivity and speed of chimera detection. Bioinformatics.

[28] Bolger A.M., Lohse M., Usadel B. (2014). Trimmomatic: A flexible trimmer for Illumina sequence data. Bioinformatics.

[29] Edgar R.C. (2013). UPARSE: Highly accurate OTU sequences from microbial amplicon reads. Nat. Methods.

[30] Quast C., Pruesse E., Yilmaz P., Gerken J., Schweer T., Yarza P., Peplies J., Glöckner F.O. (2012). The SILVA ribosomal RNA gene database project: Improved data processing and web-based tools. Nucleic Acids Res..

[31] He P., Cui W., Munir S., He P., Li X., Wu Y., Yang X., Tang P., He Y. (2019). *Plasmodiophora brassicae* root hair interaction and control by *Bacillus subtilis* XF-1 in Chinese cabbage. Biol. Control..

[32] Ahmed A., Munir S., He P., Li Y., He P., Yixin W., He Y. (2020). Biocontrol arsenals of bacterial endophyte: An imminent triumph against clubroot disease. Microbiol. Res..

[33] Srivastava R., Khalid A., Singh U., Sharma A. (2010). Evaluation of arbuscular mycorrhizal fungus, fluorescent *Pseudomonas* and *Trichoderma harzianum* formulation against *Fusarium oxysporum* f. sp. *lycopersici* for the management of tomato wilt. Biol. Control..

[34] Sarma B.K., Yadav S.K., Singh S., Singh H.B. (2015). Microbial consortium-mediated plant defense against phytopathogens: Readdressing for enhancing efficacy. Soil Biol. Biochem..

[35] Palmieri D., Vitullo D., De Curtis F., Lima G. (2017). A microbial consortium in the rhizosphere as a new biocontrol approach against *Fusarium* decline of chickpea. Plant Soil.

[36] Topalović O., Hussain M., Heuer H. (2020). Plants and associated soil microbiota cooperatively suppress plant-parasitic nematodes. Front. Microbiol..

[37] Chiaramonte J.B., Mendes L.W., Mendes R. (2021). Rhizosphere microbiome and soilborne diseases. Rhizosphere Biology: Interactions Between Microbes and Plants.

[38] Hu J., Wei Z., Friman V.P., Gu S.H., Wang X.F., Eisenhauer N., Yang T.-J., Ma J., Shen Q.-R., Xu Y.-C. (2016). Probiotic diversity enhances rhizosphere microbiome function and plant disease suppression. MBio.

[39] Kaushal M., Swennen R., Mahuku G. (2020). Unlocking the microbiome communities of banana (Musa spp.) under disease stressed (*Fusarium wilt*) and non-stressed conditions. Microorganisms.

[40] Delgado-Baquerizo M., Oliverio A.M., Brewer T.E., Benavent-González A., Eldridge D.J., Bardgett R.D., Maestre F.T., Singh B.K., Fierer N. (2018). A global atlas of the dominant bacteria found in soil. Science.

[41] Zhang T., Wang N.-F., Liu H.-Y., Zhang Y.-Q., Yu L.-Y. (2016). Soil pH is a key determinant of soil fungal community composition in the Ny-Ålesund Region, Svalbard (High Arctic). Front. Microbiol..

[42] Li C., Ahmed W., Li D., Yu L., Xu L., Xu T., Zhao Z. (2022). Biochar suppresses bacterial wilt disease of flue-cured tobacco by improving soil health and functional diversity of rhizosphere microorganisms. Appl. Soil Ecol..

[43] Wang Y., Ma Z., Wang X., Sun Q., Dong H., Wang G., Chen X., Yin C., Han Z., Mao Z. (2019). Effects of biochar on the growth of apple seedlings, soil enzyme activities and fungal communities in replant disease soil. Sci. Hortic..

[44] Wang R., Zhang H., Sun L., Qi G., Chen S., Zhao X. (2017). Microbial community composition is related to soil biological and chemical properties and bacterial wilt outbreak. Sci. Rep..

[45] He K., Yang S.-Y., Li H., Wang H., Li Z.-L. (2014). Effects of calcium carbonate on the survival of *Ralstonia solanacearum* in soil and control of tobacco bacterial wilt. Eur. J. Plant Pathol..

[46] Liu L., Huang B., Sun J., Guo S.-R., Li L.-Q., Guo H.-W. (2013). Relationship between soil microbial quantity, enzyme activity and soil fertility in hot pepper greenhouse soils of different continuous cropping years. Soil Fertil. Sci. China.

