# Supplementary material for: Microbial Consortia: An Engineering Tool to Suppress Clubroot of Chinese Cabbage by Changing the Rhizosphere Bacterial Community Composition
Source: Biology (Basel). 2022 Jun 15;11(6):918. doi: 10.3390/biology11060918 (PMC9219690; doi:10.3390/biology11060918)
Supplement: Supplementary file 1 [file biology-11-00918-s001.zip › biology-1733644-supplementary.pdf]

Microbial consortia: An engineering tool to suppress clubroot of Chinese cabbage by changing the rhizosphere bacterial community composition

Jinhao Zhang[1,2,#], Waqar Ahmed[1,2#], Zhenlin Dai[1,2#], Xinghai Zhou[1,2], Zulei He[1,2], Lanfang Wei[1,3]* and Guanghai Ji[1,2]*

[1]State Key Laboratory for Conservation and Utilization of Bio-resources in Yunnan, Yunnan Agricultural University, Kunming 650201, Yunnan, China

[2]Key Laboratory of Agro-Biodiversity and Pest Management of Ministry of Education, Yunnan Agricultural University, Kunming 650201, Yunnan, China

[3]Agricultural Foundation Experiment Teaching Center, Yunnan Agricultural University, Kunming 650201, Yunnan, China

[#]These authors contributed equally to this work.

***Correspondence:**

Guanghai Ji

jghai001@163.com

Lanfang Wei

wlfang2000@aliyun.com

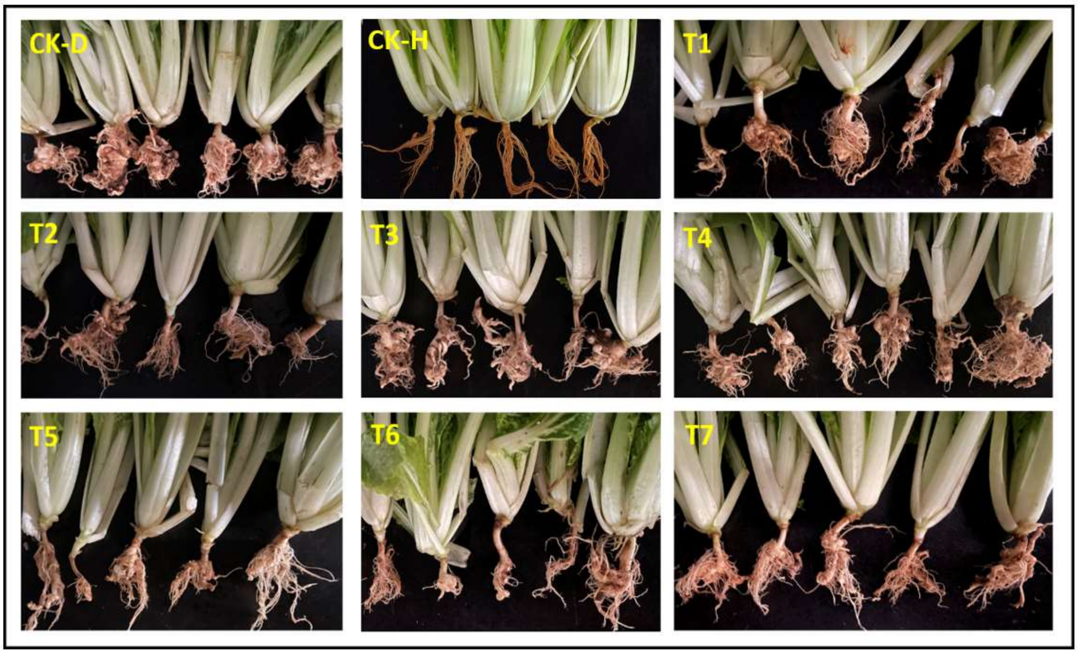

Figure S1. Effect of bacterial biocontrol strains as single strains, inter-/intra-genus, and microbial consortia on clubroot disease. Disease control **(CK-D)**, healthy control **(CK-H)**, single strain *Lysobacter antibioticus* 13-6 **(T1)**, single strain *L. capsici* ZST1-2 **(T2)**, single strain *Bacillus cereus* BT-23 **(T3)**, intra-genus co-culture of *L. antibioticus* 13-6 + *L. capsici* ZST1-2 **(T4)**, inter-genus co-culture of *L. antibioticus* 13-6 + *B. cereus* BT-23 **(T5)**, microbial consortia *B. cereus* BT-23 + *L. antibioticus* 13-6 + *L. capsici* ZST1-2 **(T6)**, and commercial fungicide Kejia **(T7)**.

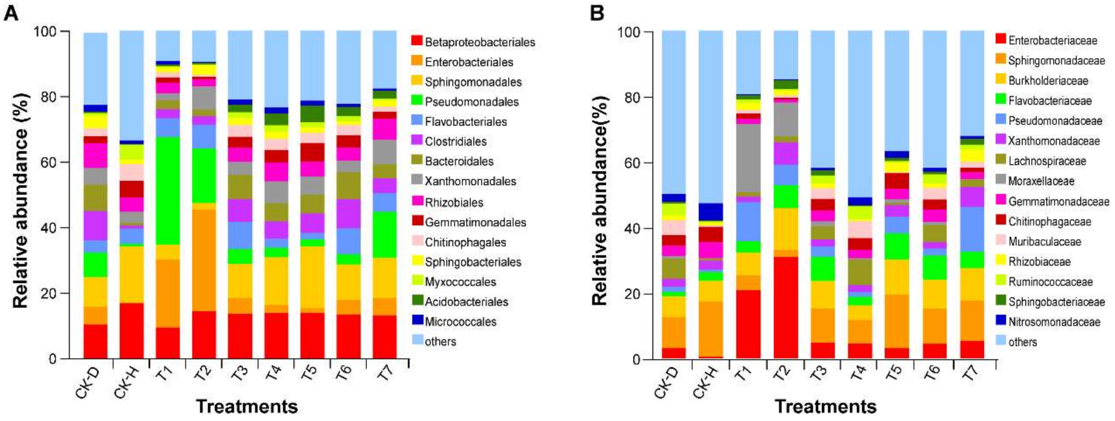

Figure S2. Relative abundacne bar plots for top 15 most abundant bacteial communities at order (A) and family (B) level. Disease control **(CK-D)**, healthy control **(CK-H)**, single strain *Lysobacter antibioticus* 13-6 **(T1)**, single strain *L. capsici* ZST1-2 **(T2)**, single strain *Bacillus cereus* BT-23 **(T3)**, intra-genus co-culture of *L. antibioticus* 13-6 + *L. capsici* ZST1-2 **(T4)**, inter-genus co-culture of *L. antibioticus* 13-6 + *B. cereus* BT-23 **(T5)**, microbial consortia *B. cereus* BT-23 + *L. antibioticus* 13-6 + *L. capsici* ZST1-2 **(T6)**, and commercial fungicide Kejia **(T7)**.

Table S1. Effect of bacterial biocontrol strains on the disease incidence, disease index, control effect, and soil pH of cabbage clubroot.

Treatment	Disease incidence (%)	Disease index	Control effect (%)	pH
CK-D	93.33±3.33a	76.28±9.43a	-	4.20±0.06b
CK-H	8.89±1.92e	14.29±2.57f	81.27±3.67a	4.45±0.02ab
T1	77.78±11.70b	37.96±12.57cd	50.23±9.09d	4.42±0.34ab
T2	75.56±6.94bc	31.51±3.13de	58.69±1.67c	4.46±0.04ab
T3	77.78±8.39b	50.20±9.43b	34.19±2.63f	4.27±0.16ab
T4	72.22±6.94bc	41.54±3.15c	45.54±3.29e	4.63±0.58ab
T5	68.89±6.69bcd	31.79±10.99de	58.32±8.07c	4.65±0.06ab
T6	56.67±8.82d	26.10±7.12e	65.78±5.83b	4.73±0.12a
T7	63.33±8.82cd	32.30±7.85d	57.66±6.65c	4.73±0.08a

Disease control **(CK-D)**, healthy control **(CK-H)**, single strain *Lysobacter antibioticus* 13-6 **(T1)**, single strain *L. capsici* ZST1-2 **(T2)**, single strain *Bacillus cereus* BT-23 **(T3)**, intra-genus co-culture of *L. antibioticus* 13-6 + *L. capsici* ZST1-2 **(T4)**, inter-genus co-culture of *L. antibioticus* 13-6 + *B. cereus* BT-23 **(T5)**, microbial consortia *B. cereus* BT-23 + *L. antibioticus* 13-6 + *L. capsici* ZST1-2 **(T6)**, and commercial fungicide Kejia **(T7)**. Significant difference among treatments is shown by different small letters within a column according to Duncan's multiple range test at $p < 0.05$.

Table S2. Effect of bacterial biocontrol strains on yield of Chinese cabbage.

Treatment	Yield (Kg/Acre)	Rate of growth (%)
CK-D	7165.45±1068.49c	-
CK-H	16875.33±929.91a	135.51
T1	13167.53±899.23b	83.76
T2	14015.27±545.21b	95.66
T3	12748.88±174.78b	77.92
T4	13020.15±871.22b	81.71
T5	16070.65±764.12a	124.28
T6	17662.49±1545.05a	146.50
T7	16097.64±1746.36a	124.66

Disease control **(CK-D)**, healthy control **(CK-H)**, single strain *Lysobacter antibioticus* 13-6 **(T1)**, single strain *L. capsici* ZST1-2 **(T2)**, single strain *Bacillus cereus* BT-23 **(T3)**, intra-genus co-culture of *L. antibioticus* 13-6 + *L. capsici* ZST1-2 **(T4)**, inter-genus co-culture of *L. antibioticus* 13-6 + *B. cereus* BT-23 **(T5)**, microbial consortia *B. cereus* BT-23 + *L. antibioticus* 13-6 + *L. capsici* ZST1-2 **(T6)**, and commercial fungicide Kejia **(T7)**. Significant difference among treatments is shown by different small letters within a column according to Duncan's multiple range test at $p < 0.05$.

Table S3. Effect of single strains, intra/inter-genus co-culture, and microbial consortia on alpha diversity of bacterial community associated with the rhizosphere of Chinese cabbage (±SEM, n = 3/treatment).

Treatment	Chao1	Goods_coverage	Shannon	Observed_species	Simpson	PD_whole_tree
CK-D	2694.36±159.32c	0.98±0a	6.32±0.24d	1862.87±108.13e	0.95±0.01b	72.13±3.26e
CK-H	3995.20±169.91a	0.98±0a	9.53±0.27a	3235.27±211.87a	1.00±0a	108.94±7.04a
T1	3433.88±189.53ab	0.98±0a	8.70±1.08a	2632.20±201.51c	0.98±0.02a	92.95±5.00a
T2	3453.39±143.99ab	0.98±0a	8.84±0.33ab	2602.90±126.72c	0.99±0a	91.84±3.61bc
T3	3089.13±255.56b	0.98±0a	8.06±0.57c	2310.70±233.38d	0.98±0.01a	83.70±5.85d
T4	3619.18±207.86a	0.98±0a	9.46±0.18a	2858.70±139.39b	1.00±0a	100.42±3.76a
T5	3411.68±112.85ab	0.98±0a	9.13±0.27ab	2684.27±117.31c	0.99±0.01a	94.43±2.49b
T6	3487.64±127.3ab	0.98±0a	9.23±0.06a	2655.10±87.67c	0.99±0a	94.33±2.58b
T7	3467.79±257.75ab	0.98±0a	8.50±0.46b	2580.17±214.62cd	0.99±0a	90.37±5.30c

Disease control **(CK-D)**, healthy control **(CK-H)**, single strain *Lysobacter antibioticus* 13-6 **(T1)**, single strain *L. capsici* ZST1-2 **(T2)**, single strain *Bacillus cereus* BT-23 **(T3)**, intra-genus co-culture of *L. antibioticus* 13-6 + *L. capsici* ZST1-2 **(T4)**, inter-genus co-culture of *L. antibioticus* 13-6 + *B. cereus* BT-23 **(T5)**, microbial consortia *B. cereus* BT-23 + *L. antibioticus* 13-6 + *L. capsici* ZST1-2 **(T6)**, and commercial fungicide Kejia **(T7)**. Significant difference among treatments is shown by different small letters within a column according to *t*-test *at* $p < 0.05$.

Table S4. Relative abundance of 10 most dominant phyla under different experimental conditions (±SEM, n = 3/treatment).

Phylum	CK-D	CK-H	T1	T2	T3	T4	T5	T6	T7	Average (%)
Proteobacteria	61.04±1.38abc	71.75±9.83a	48.74±3.10d	57.17±5.83bcd	64.38±3.87ab	48.48±4.65d	53.69±2.5bcd	50.94±3.46cd	64.01±3.81ab	57.80
Bacteroidetes	16.84±2.39a	17.10±9.35a	20.64±1.64a	18.78±3.70a	18.64±2.46a	21.03±2.18a	16.85±4.09a	18.17±2.38a	19.23±2.66a	18.59
Firmicutes	1.73±0.25d	4.19±0.28cd	11.99±1.08a	7.61±1.37bc	6.26±0.41c	13.73±1.94a	7.88±0.94bc	10.80±0.64ab	4.95±2.63cd	7.68
Actinobacteria	8.89±1.14a	3.49±0.55c	9.64±2.43a	7.55±0.46ab	5.01±1.18bc	7.62±1.12ab	8.85±0.74a	8.87±1.65a	5.72±0.39bc	7.29
Gemmatimonadetes	5.83±1.23a	1.38±0.41d	3.68±0.88bc	3.25±0.76bc	2.05±0.59cd	3.60±0.30bc	4.55±0.96ab	3.73±0.47bc	2.88±0.32bcd	3.44
Acidobacteria	3.08±0.82bc	0.99±0.28d	1.96±0.43cd	3.53±0.63bc	2.24±1.4cd	1.98±0.23cd	5.71±1.74a	4.48±0.28ab	1.77±0.57cd	2.86
Cyanobacteria	0.14±0.03e	0.38±0.01de	1.60±0.07a	0.86±0.15bc	0.63±0.12bcd	1.67±0.34a	0.96±0.14b	1.36±0.12a	0.50±0.24cde	0.90
Nitrospirae	1.62±0.44a	0.15±0.06c	0.40±0.26bc	0.51±0.1bc	0.26±0.05bc	0.59±0.13bc	0.59±0.19bc	0.63±0.12b	0.40±0.11bc	0.57
Verrucomicrobia	0.14±0.04cd	0.04±0.01d	0.31±0.05ab	0.16±0.05c	0.11±0.02cd	0.34±0.09a	0.19±0.02c	0.21±0.05bc	0.10±0.05cd	0.18
Low Abundance	0.69±0.26abc	0.53±0.24c	1.04±0.18a	0.59±0.15bc	0.42±0.06c	0.95±0.12ab	0.73±0.05abc	0.8±0.12abc	0.45±0.18c	0.69

Disease control **(CK-D)**, healthy control **(CK-H)**, single strain *Lysobacter antibioticus* 13-6 **(T1)**, single strain *L. capsici* ZST1-2 **(T2)**, single strain *Bacillus cereus* BT-23 **(T3)**, intra-genus co-culture of *L. antibioticus* 13-6 + *L. capsici* ZST1-2 **(T4)**, inter-genus co-culture of *L. antibioticus* 13-6 + *B. cereus* BT-23 **(T5)**, microbial consortia *B. cereus* BT-23 + *L. antibioticus* 13-6 + *L. capsici* ZST1-2 **(T6)**, and commercial fungicide Kejia **(T7)**. Significant difference among treatments is shown by different small letters within a column according to t-test at $p < 0.05$.

Table S5. Relative abundance of 20 most dominant genera in rhizosphere soil under different experimental conditions (±SEM, n = 3/treatment).

Genus	CK-D	CK-H	T1	T2	T3	T4	T5	T6	T7	Average (%)
Sphingomonas	14.02±0.10a	3.70±0.83e	8.28±1.44cd	10.67±1.25abc	5.66±1.77de	6.73±1.17cde	13.54±3.61ab	10.94±0.72abc	9.36±3.15bcd	9.21
Pseudomonas	2.24±2.20c	12.7±3.90a	4.14±1.94bc	8.53±4.14ab	10.89±1.03a	3.90±2.87bc	2.53±0.54c	2.70±0.64c	11.07±2.69a	6.52
Flavobacterium	5.31±2.41a	8.71±6.42a	3.45±1.92a	6.09±3.00a	7.38±4.00a	2.59±0.20a	3.61±3.05a	4.53±2.40a	7.49±4.48a	5.46
Acinetobacter	0.38±0.46b	11.41±8.38a	0.93±0.47b	0.97±0.58b	4.42±3.48b	1.24±0.67b	0.79±0.52b	0.70±0.28b	2.25±1.52b	2.57
Citrobacter	0.06±0.03b	5.62±3.05a	0.47±0.11b	0.92±0.99b	3.56±3.46ab	1.01±0.56b	0.23±0.08b	0.34±0.08b	1.97±1.02b	1.58
Allorhizobium-Neorhizobium-Pararhizobium-Rhizobium	1.02±0.86b	2.06±0.72ab	1.92±0.95ab	1.48±0.42ab	1.23±0.2ab	0.79±0.46b	0.96±0.27b	1.09±0.26ab	2.48±0.67a	1.45
Massilia	1.05±0.31ab	1.07±0.09ab	0.88±0.32b	1.87±1.29ab	0.92±0.48b	0.79±0.32b	1.62±0.5ab	2.02±0.83ab	2.58±1.01a	1.42
Stenotrophomonas	0.31±0.30a	4.45±4.89a	0.84±0.64a	1.86±1.31a	1.75±0.20a	0.90±0.69a	0.39±0.23a	0.60±0.22a	1.65±0.38a	1.42
Gemmatimonas	1.14±0.06bcd	0.68±0.18d	1.13±0.29bcd	1.55±0.39bc	1.04±0.38cd	1.07±0.19bcd	2.51±0.52a	1.76±0.28b	1.26±0.08bcd	1.35
Lysobacter	0.46±0.07c	1.80±0.12a	1.49±0.13ab	1.19±0.18bc	1.18±0.09bc	1.37±0.11abc	1.47±0.51ab	1.79±1.21a	1.38±0.27ab	1.35
Bacteroides	0.21±0.08e	0.53±0.02de	1.62±0.20ab	1.18±0.29abc	0.86±0.1bcd	2.08±0.42a	1.26±0.07abc	1.37±0.04ab	0.72±0.42cde	1.09
Bifidobacterium	0.14±0.03d	0.64±0.01cd	1.71±0.17a	0.88±0.16bc	1.10±0.51bc	1.82±0.31a	0.86±0.29bc	1.42±0.06ab	0.55±0.27cd	1.01
Variovorax	0.91±0.46a	0.95±0.07a	0.77±0.20a	1.17±0.20a	0.91±0.36a	0.55±0.22a	1.30±0.52a	1.29±0.23a	1.20±0.47a	1.01
Chryseobacterium	0.31±0.37b	1.91±1.11a	0.59±0.38b	0.85±0.31ab	1.50±0.09ab	1.02±0.92ab	0.62±0.13b	0.75±0.37ab	1.26±0.14ab	0.98
Solanum_torvum	0.08±0.02e	0.41±0.03de	1.62±0.07a	0.87±0.15bc	0.67±0.15bcd	1.76±0.36a	0.97±0.15b	1.42±0.11a	0.51±0.26cd	0.92
Altererythrobacter	1.78±0.12a	0.37±0.00d	1.13±0.29b	0.95±0.08b	0.45±0.12cd	0.83±0.16bc	0.95±0.16b	0.79±0.16bcd	0.84±0.41bc	0.90
Escherichia-Shigella	0.22±0.08d	0.39±0.15cd	1.41±0.19a	0.88±0.21b	0.59±0.02c	1.69±0.23a	0.77±0.07b	1.43±0.06a	0.49±0.18c	0.87
Acidovorax	0.72±0.62c	1.57±0.63b	0.55±0.32cd	0.49±0.32cd	0.75±0.40c	0.46±0.29cd	0.18±0.09d	0.35±0.17d	2.58±0.72a	0.85
Sphingobacterium	0.13±0.12d	1.35±0.64ab	0.96±1.20c	0.78±0.61b	1.78±0.51a	0.24±0.19d	0.22±0.14d	0.21±0.05d	1.35±0.20ab	0.78
Bacillus	0.18±0.08a	0.17±0.14a	0.13±0.07a	0.17±0.06a	0.19±0.12a	0.11±0.01a	0.17±0.03a	0.20±0.08a	0.14±0.04a	0.16

Disease control **(CK-D)**, healthy control **(CK-H)**, single strain *Lysobacter antibioticus* 13-6 **(T1)**, single strain *L. capsici* ZST1-2 **(T2)**, single strain *Bacillus cereus* BT-23 **(T3)**, intra-genus co-culture of *L. antibioticus* 13-6 + *L. capsici* ZST1-2 **(T4)**, inter-genus co-culture of *L. antibioticus* 13-6 + *B. cereus* BT-23 **(T5)**, microbial consortia *B. cereus* BT-23 + *L. antibioticus* 13-6 + *L. capsici* ZST1-2 **(T6)**, and commercial fungicide Kejia **(T7)**. Significant difference among treatments is shown by different small letters within a column according to *t*-test at $p < 0.05$.
